# Wernicke Encephalopathy Associated With Semaglutide Use

**DOI:** 10.7759/cureus.61783

**Published:** 2024-06-06

**Authors:** Krishna Sheth, Elizabeth Garza, Ajith Saju, Natasha Nazir, Aditya Agarwal

**Affiliations:** 1 Internal Medicine, Garnet Health Medical Center, Middletown, USA; 2 Internal Medicine, Touro College of Osteopathic Medicine, Middletown, USA

**Keywords:** non-alcoholic wernicke encephalopathy, encephalopathy, wernicke encephalopathy, semaglutide, ozempic

## Abstract

A patient presenting to the emergency room with neurological symptoms is more commonly found to have manifestations of stroke, transient ischemic attack, or nervous system injury. Alcoholic Wernicke encephalopathy (WE) is also another common manifestation of neurological dysfunction; however, the prevalence of non-alcoholic WE is relatively uncommon. We discuss a 37-year-old male who presented to the ED with dysphagia, slurred speech, word-finding difficulty, and restricted extraocular movements from non-alcoholic WE in the setting of semaglutide use.

## Introduction

Non-alcoholic Wernicke encephalopathy (WE) is a relatively rare diagnosis with an estimated prevalence of 0.04-2.8%, including autopsy studies [1]. WE is a medical emergency characterized by the abrupt emergence of neurological deficits, most commonly including horizontal gaze-evoked nystagmus [1,2]. Sometimes patients with WE present with the triad of ataxia, altered mental status, and ocular dysfunction [1].

Deficiency of thiamine, also known as vitamin B1, is the primary cause of WE. It can commonly arise from prolonged fasting, bariatric surgery, and anorexia nervosa [2]. Inadequate nutrition can also result in vitamin deficiencies, namely thiamine, which can cause Wernicke syndrome [2]. Normally, thiamine helps the brain convert sugar into energy; however, when the vitamin is deficient, patients present with encephalopathy [2].

There are numerous causes of thiamine deficiency, one of which includes the popular weight-loss medication semaglutide [3]. Semaglutide is a glucagon-like peptide-1 receptor agonist (GLP-1 RA) that mimics the hormone released by the gut after eating [3]. When this receptor is activated, the hunger response decreases. Hence, it is crucial to establish a link between possible vitamin deficiencies and appetite-suppressing medications.

## Case presentation

A 37-year-old male with a past medical history of anxiety, chronic obstructive pulmonary disease, diabetes mellitus, hyperlipidemia, hypertension, major depressive disorder, polysubstance abuse, and thyroid disease was brought into the ED from a correctional facility with dysphagia, slurred speech, word-finding difficulty, a severe left-sided headache, and restricted extraocular movements. In the ED, he had normal vital signs, left-sided facial droop, and over 70 lbs weight loss. The dysphagia and slurred speech started two weeks prior to arrival. He described that he recently started taking semaglutide (Ozempic) for the past several months; however, it made him nauseous. He endorsed several episodes of emesis and decreased appetite. He denied any tobacco smoking or alcohol use but admitted to smoking K2 synthetic marijuana two weeks prior to arrival. On the physical exam, he had slurring of speech, weakness, and anxiety. On a visual exam, he had abnormal extraocular motions and bilateral esotropia and was admitted to the hospital to rule out a cerebrovascular accident given the patient's symptoms and presentation. A CT of the head/brain without contrast revealed no intracranial hemorrhage, acute infarct, or mass lesions. A CT angiography of the head and neck revealed no aneurysms, vascular malformations, or stenosis of the visualized intracranial circulation. Prior to arrival, medications included an albuterol inhaler, escitalopram 10 mg, a fluticasone-salmeterol inhaler, folic acid 1 mg, indomethacin 25 mg, levothyroxine 75 mcg, lisinopril 20 mg, and semaglutide 2 mg. In the ED, he started on aspirin 81 mg and atorvastatin 80 mg. Neurology was consulted.

**Figure 1:**
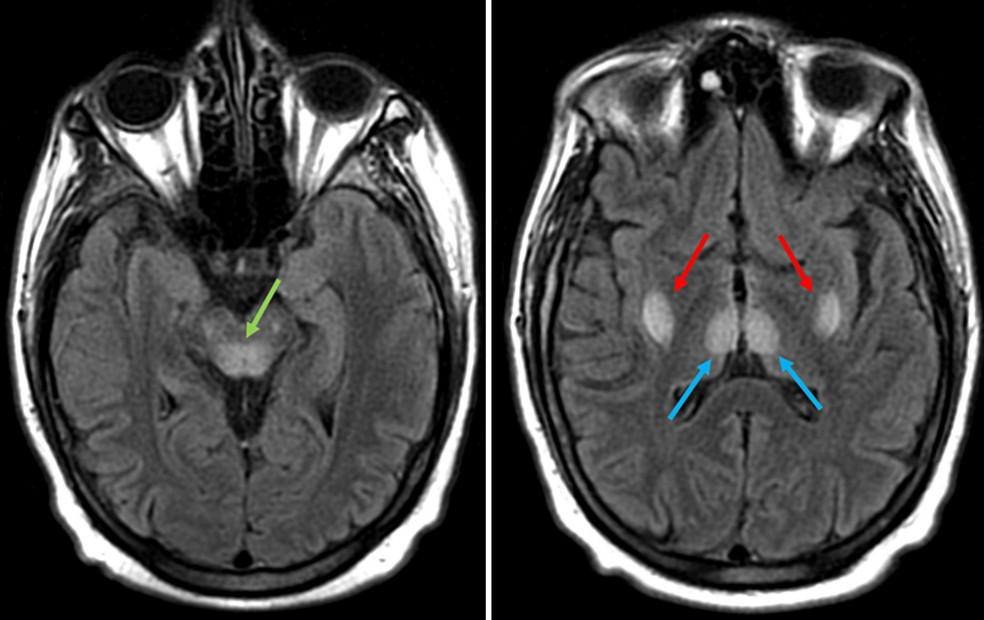
MRI brain without contrast revealed symmetrical signal abnormalities involving the basal ganglia (red arrow), thalamus (blue arrow), and brainstem (green arrow) MRI: magnetic resonance imaging

MRI of the brain revealed symmetrical signal abnormalities involving the basal ganglia, thalamus, and brain that indicated differentials of toxic encephalopathy concerning WE or hypoxia, as seen in Figure 1. The electrocardiogram showed normal sinus rhythm with borderline QT segment prolongation, and telemetry monitoring did not show arrhythmia.

Furthermore, the echocardiogram showed normal systolic and diastolic function without any thrombus or valvular dysfunction. Toxicology screening and Lyme disease results were unremarkable. The speech and language pathology (SLP) team was consulted for sustained dysphasia. X-ray video speech evaluation showed aspiration with thin liquids. The patient was given IV thiamine 500 mg and folic acid 1 mg daily. After the initiation of treatment, his slurred speech returned, and his left-sided facial weakness and headaches resolved. For the sustained nystagmus, an eye patch was used, with an improvement in left eye tracking. Dysphagia continued to improve throughout the stay. The risk of silent aspiration with thin liquids was a concern prior to discharge, which the SLP addressed and recommended transfer to a center for continuing dysphagia therapy.

The patient was treated with intravenous thiamine 500 mg three times a day for three days. Per a neurology consult, ophthalmoplegia and associated symptoms were due to WE. Given the improvement with the thiamine, a lumbar puncture and a video-electroencephalogram were not necessary.

## Discussion

WE, which arises due to thiamine deficiency, is typically characterized by ataxia, nystagmus, and cognitive dysfunction [3]. There are numerous causes of thiamine deficiency, which include the popular weight-loss medication semaglutide. If thiamine deficiency is left untreated, it can have fatal complications. Hence, it is crucial to establish a link between possible vitamin deficiencies and appetite-suppressing medications. When left untreated, WE can transition to Korsakoff syndrome in 80% of cases [2]. Symptoms of Korsakoff syndrome include memory impairment, confabulation, and mood disturbance. On gross pathology of the brain, about 50% of WE cases will have bilateral symmetric lesions of grayish discoloration, congestion, and pinpoint hemorrhages [2]. Per the literature review, one of the few documented instances of a prisoner experiencing WE during incarceration resulted from a hunger strike [4]. Inadequate nutrition can result in vitamin deficiencies, namely thiamine, and result in Wernicke syndrome [3].

A relatively frequently prescribed pharmaceutical treatment for weight loss and diabetes mellitus type 2 is semaglutide [3]. It is also more commonly known as the Ozempic. Semaglutide is a glucagon-like peptide agonist [3]. It has shown efficacy in glucose control for diabetes mellitus type 2 patients by increasing insulin secretion, lowering glucagon release, and slowing gastric emptying [5]. Nausea, vomiting, and decreased gastric emptying are the most common side effects of Ozempic [5]. Furthermore, it may also increase satiety and decrease appetite, as witnessed in our patient. The decrease in food intake can prevent a patient from receiving vital nutrients that sustain neurological function. In a recent case report, a patient was described as having presented to the ED with bidirectional nystagmus, gait ataxia, and altered mental status, which was later diagnosed as WE [6]. In our case, the patient had recently lost 70 lbs after three months of semaglutide use, which significantly impacted his appetite. Therefore, potential issues previously stated, such as decreased food tolerance or suppressed intake, may be associated with semaglutide usage. The lack of emphasis on examining these consequences when instructing individuals about appetite-suppressing medications proves to be worrisome.

## Conclusions

This case seeks to emphasize the significance of delivering thorough education on sustaining adequate vitamin intake despite diminished appetite, especially while on GLP-1 RA such as Ozempic. Lack of emphasis on explaining these consequences when instructing patients about appetite-suppressing medication can result in detrimental complications and unnecessary hospitalizations. Proper education on medication use and potential side effects is crucial to allowing patients to be more wary of symptoms.
